# Improved method for assessing iron stores in the bone marrow

**DOI:** 10.1136/jcp.2009.064451

**Published:** 2009-07-20

**Authors:** K S Phiri, J C J Calis, D Kachala, E Borgstein, J Waluza, I Bates, B Brabin, M Boele van Hensbroek

**Affiliations:** 1 Malawi–Liverpool–Wellcome Trust Clinical Research Programme, College of Medicine, Blantyre, Malawi; 2 Emma Children’s Hospital AMC, University of Amsterdam, Netherlands; 3 Department of Surgery, College of Medicine, Blantyre, Malawi; 4 Liverpool School of Tropical Medicine, Liverpool, UK

## Abstract

**Background:**

Bone marrow iron microscopy has been the “gold standard” method of assessing iron deficiency. However, the commonly used method of grading marrow iron remains highly subjective.

**Aim:**

To improve the bone marrow grading method by developing a detailed protocol that assesses iron in fragments, in macrophages around fragments and in erythroblasts.

**Methods:**

A descriptive study of marrow aspirates of 303 children (aged 6–60 months) with severe anaemia and 22 controls (children undergoing elective surgery) was conducted at hospitals in southern Malawi (2002–04).

**Results:**

Using an intensive marrow iron grading method, 22% and 39% of cases and controls had deficient iron stores, and 40% and 46% had functional iron deficiency, respectively. Further evaluation of the iron status classification by the intensive method showed that functional iron deficiency was associated with significantly increased C-reactive protein concentrations (126.7 (85.6) mg/l), and iron stores deficiency with significantly increased soluble transferrin receptor concentrations (21.7 (12.5) μg/ml).

**Conclusions:**

Iron assessment can be greatly improved by a more intense marrow examination. This provides a useful iron status classification which is of particular importance in areas where there is a high rate of inflammatory conditions.

Functional iron deficiency develops when normal physiological systems for transporting iron to target tissues are impaired in the presence of satisfactory iron stores.^1^ This is commonly caused by cytokines released during an acute phase response to infection, leading to impaired erythropoiesis and later anaemia, usually termed “anaemia of inflammation”.^2^

Although mass spectrometry has been recently used to give a definitive determination of iron in tissue,^3^ ^4^ microscopic examination of Prussian blue-stained bone marrow aspirate has been considered the practical “gold standard” for determining iron depleted states.^5^ ^6^ Conventionally, iron has been primarily assessed in marrow fragments which represent iron stores in the form of hemosiderin.^7^ ^8^ Although some studies have shown a reasonable correlation between histological iron grading and chemical iron concentration in bone marrow,^8^ ^9^ others have not, raising questions about the validity of the histological grading.^10^

Iron visualised in marrow fragments is from a meshwork of reticular cells which are usually undistinguishable. However, single “loose” macrophages may be inspected for iron and it is hypothesised to be particularly important when iron in fragments is absent and may signify the lowest level of iron stores depletion.

In areas where there is a high prevalence of inflammatory conditions, functional iron deficiency commonly occurs. Erythroblast iron may be indicative of cellular iron utilisation and decreased in functional iron deficiency^11^; however there has been little research on the use of erythroblast iron as a marker of cellular iron availability.^12^ Furthermore, in malaria endemic areas, interpretation of iron status may be confounded by the presence of hemozoin.^13^ ^14^

Recent studies have suggested that it may be detrimental to mass treat children with iron,^15^ so it is important to be able to identify children with iron stores deficiency. The aim of the present study was to determine if a more intensive bone marrow classification can distinguish iron deficiency states in severely anaemic children and their controls living in a malaria endemic area.

## METHODS

This was a descriptive study which was part of a large case–control study investigating the aetiology of severe anaemia in Malawi. Children were recruited between July 2002 and July 2004 at Queen Elizabeth Central hospital in Blantyre and Chikwawa District hospital.^16^

All children who presented to hospital with severe anaemia (haemoglobin of less than 5.0 g/dl and no history of being transfused in the preceding four weeks), aged 6–60 months, were eligible for recruitment as cases. A group of children undergoing elective operations with no obvious signs of infection and within the same age range as cases were recruited as “normal” controls. Samples of venous blood and bone marrow aspirate were collected from cases and controls under anaesthesia from either the anterior or posterior iliac crest. The first few drops of a bone marrow aspirate were collected in an EDTA tube for smear preparation.^16^

Written informed consent was obtained from the guardians of the children and the study was approved by the ethics committees of the University of Malawi and the Liverpool School of Tropical Medicine, UK.

### Bone marrow smear preparation

Bone marrow smears were air-dried before being fixed in methanol for 5 minutes. Iron staining was done at the Wellcome Trust laboratories, Blantyre, using a commercial kit and according to methods recommended by the manufacturer (HematoGnost Fe, Darmstadt, Germany). Positive controls were included in each batch of slides.

### Bone marrow smear iron grading

Bone marrow smears were graded by the conventional Gale’s method and by a new more intensive grading method.

Marrow smears were first assessed by one of the authors (KP) according to Gale’s histological grading method^8^ which assesses only marrow fragments (table 1). In order to reduce subjectivity, predefined descriptions and sample illustrations of each iron grade were used to grade fragments of all marrow smears. Only iron smears with at least seven fragments were assessed.^17^ Deficiency of iron stores was defined as an iron grade of none (grade 0) or very slight (grade 1).

**Table 1:** Histological grading for bone marrow iron status according to Gale *et al*^8^

Grade 0	None	No visible iron under high power magnification (×1000)
Grade 1	Very slight	Small iron particles just visible in few reticulum cells under high power magnification (×1000)
Grade 2	Slight	Small, sparsely distributed iron particles just visible under low power magnification (×100)
Grade 3	Moderate	Numerous small iron particles present in reticulum cells throughout the marrow fragment (×100)
Grade 4	Moderate heavy	Larger iron particles throughout the fragment with tendency to aggregate into clumps (×100)
Grade 5	Heavy	Dense, large clumps of iron throughout the fragment (×100)
Grade 6	Very heavy	Very large deposits of iron, both intra- and extra-cellular, obscuring cellular detail in the fragment (×100)

**Figure 1:**
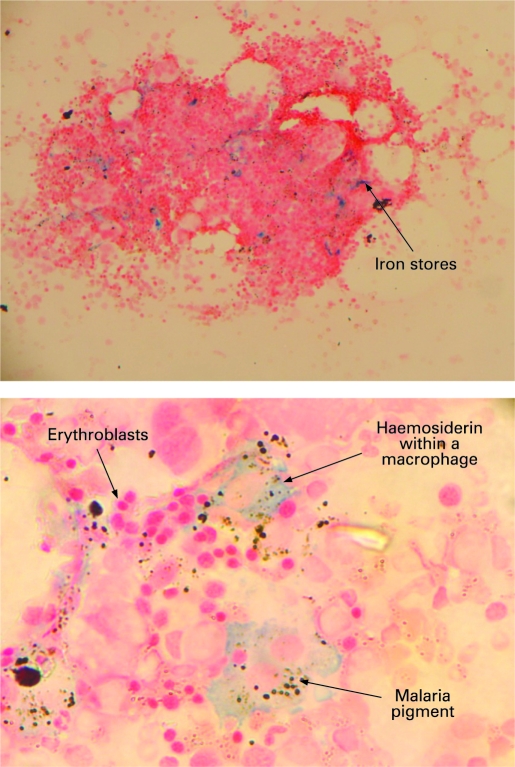
Bone marrow fragment showing iron deposits at ×400 magnification (top), and ×1000 magnification showing erythroblasts, iron and malaria pigment within macrophages (bottom).

All marrow smears were then systematically assessed using an intensive histological grading method in which iron is assessed in three sites—the fragments (as in Gale’s method), macrophages and erythroblasts. Iron assessed in the fragments and macrophages represented iron stores while iron in the erythroblast represented utilisable iron. Additionally, 20 fields around and behind the fragments were examined at high power (×1000) and all macrophages in these fields were examined for the presence of iron (fig 1). At high power magnification (×1000), 100 erythroblasts were examined and the percentage containing iron granules in their cytoplasm (ie, sideroblasts) were enumerated. Erythroblast iron deficiency was defined when <30% of erythroblasts had visible iron granules.^18^

Results of iron smear assessment using the intensive histological grading method were interpreted as *normal status* (normal iron stores and normal erythroblast iron); *functional iron deficiency* (normal iron stores and deficient erythroblast iron); *iron stores deficiency* (depleted iron stores and normal erythroblast iron); and *combined functional iron and iron stores deficiency* (depleted iron stores and deficient erythroblast iron; table 2).

**Table 2:** Classification of iron status using the intensive grading method

Iron detected in:			Iron status category
Fragment*	Macrophage†	Erythroblast‡	
Present	Present	Present	Normal
Present	**–**	Present	
Present	Present	**–**	Functional iron deficiency
Present	**–**	**–**	
**–**	Present	Present	Iron stores deficiency
**–**	**–**	Present	
**–**	Present	**–**	Functional and iron stores deficiency
**–**	**–**	**–**	

*Positive fragment iron: fragment grade ⩾2.

†Positive macrophage iron: iron present in reticular cell.

‡Positive erythroblast iron: iron present in >30% of erythroblasts.

### Other laboratory tests

Bone marrow iron status assessment was compared to peripheral blood iron markers from samples taken at the same time as the bone marrow aspirate. Haemoglobin was measured using a Coulter counter machine (Beckman Coulter, Durban, South Africa). Ferritin, a measure of iron stores deficiency, was determined using the electrochemiluminescence immunoassay (Modular Analytics E170, Roche Diagnostics, Switzerland), and soluble transferrin receptor (sTfR) levels, a measure of cellular iron need, using ELISA (Ramco Laboratories, Texas, USA). Immunoturbidimetric assay (Modular P800, Roche Diagnostics, Switzerland) was used to determine C-reactive protein (CRP) levels (measure of inflammation) in blood.

### Statistical analysis

Data were double entered using Microsoft Access and Microsoft Excel. All data were exported to SPSS for Windows for analysis. Difference in means was evaluated using Student’s t test and difference in proportions, using the χ^2^ test. Odds ratios (OR) and their 95% CI were used to measure association between categorical variables.

## RESULTS

A total of 381 cases and 23 controls were recruited. Cases had an average age of 1.7 years (SD 1.1), and 46.7% (178/381) were male; controls had an average age of 1.8 years (SD 0.9) and 86.4% (19/22) were male. Death in-hospital occurred in 6.3% (24/381) of cases, with no deaths in controls (table 3).

**Table 3:** Baseline characteristics of cases and controls

Characteristic	Cases	Controls
Recruited	381	23
Age (years)*	1.7 (1.1) (371)	1.8 (0.9) (21)
Hb (g/dl)*	3.6 (0.8) (381)	9.9 (1.8) (23)
Male	178/381 (46.7%)	19/22 (86.4%)
History of previous transfusion	57/378 (15.1%)	0/23 (0%)
Wasted†	53/330 (16.1%)	3/16 (18.8%)
Stunted‡	176/331 (53.2%)	7/17 (41.2%)
Malaria§	226/380 (59.5%)	0/23 (0%)
HIV¶	45/357 (12.6%)	0/9 (0%)
Bacteraemia**	54/359 (15.0%)	1/14 (7.1%)

*Mean (SD) (total no).

†Z-score <−2 weight-for-height.

‡Z-score <−2 height-for-age.

§Presence of *Plasmodium falciparum* asexual parasites in blood.

¶Positive for two rapid tests according to WHO guidelines. Discordant results and reactive results in children less than 18 month were resolved by PCR.^19^

**Presence of pathogenic bacteria using an automated BacT/Alert system (BioMerieux, Missouri, USA) and cultured for 5 and 56 days for routine pathogens and mycobacteria, respectively.

**Figure 2:**
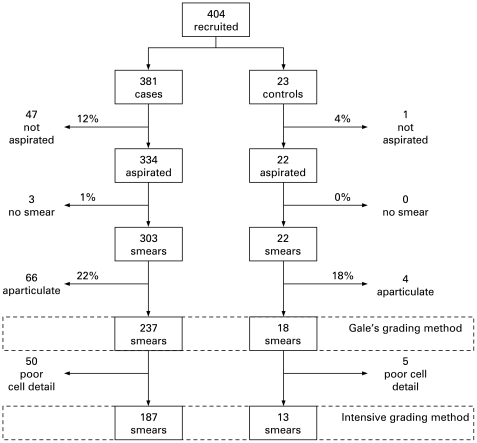
Flow chart showing the number of children recruited and the number of bone marrow samples finally assessed using both Gale’s and intensive histological grading methods.

Of a total of 381 cases, 334 bone marrow aspirations were attempted, from which 303 marrow smears were prepared (fig 2). Forty-seven (12%) cases did not have a bone marrow aspiration for the following reasons: refusal by guardian, child being too sick, unsuccessful aspiration. Twenty-two of 23 (96%) controls had a bone marrow aspirate collected and smear prepared. Therefore 303 case smears and 22 control smears were available for assessment.

### Gale’s grading method

From the total smears considered for assessment, 66 of 303 (22%) cases and 4 of 22 (18%) controls were not assessed because they contained inadequate bone marrow fragments for proper assessment. Assessment of the remaining smears showed that iron deficiency was present among 33.8% (80/237) of cases and 61.1% (11/18) of controls. According to conventional grading, iron stores deficiency was more frequent among the controls than cases, but this difference was not significant (OR  = 1.8, 95% CI 0.8 to 4.25).

### Intensive grading method

Staining quality was adequate to enable assessment of macrophage and erythroblast iron in 79% of cases (187/237) and 72% of controls (13/18). Cases and controls were classified into different iron status categories depending on iron assessment in fragment, macrophage and erythroblast as shown in table 4. Functional iron deficiency was the most common iron status category among both cases (39.6%; 74/187) and controls (46.2%; 6/13). Iron stores deficiency was less frequent among cases (21.9%; 41/187) than controls (38.5%; 5/13; p = 0.2).

**Table 4:** Bone marrow iron status category results using the ntensive grading method

Iron status category	Cases (n = 187)	Controls (n = 13)	OR* (95% CI)	p Value
Functional iron deficiency (%)	39.6	46.2	0.7 (0.2 to 2.9)	0.9
Normal iron (%)	31.0	7.7	5.4 (0.8 to 234.6)	0.1
Iron stores deficiency (%)	21.9	38.5	0.5 (0.1 to 1.9)	0.2
Functional and stores deficiency (%)	7.5	7.7	1.0 (0.1 to 44.4)	0.9

*OR  =  [odds in cases]/[odds in controls].

**Figure 3:**
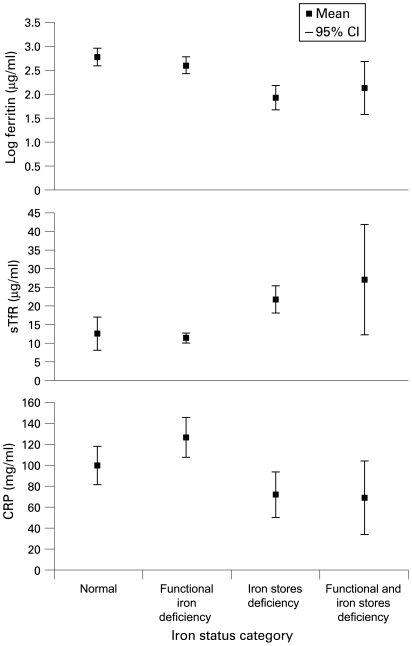
Error bar graphs for mean log ferritin, soluble transferrin receptor (sTfR) and C-reactive protein (CRP) concentrations for the different iron status classes using the intensive grading method.

Categories of iron status classified by the intensive grading method were compared with values for peripheral blood markers of iron stores (fig 3). Low levels of ferritin and high levels of sTfR signify deficiency of iron stores in the absence of inflammation.^20^ ^21^ Mean ferritin concentration was lower in children with deficiency of iron stores (1.9 (SD 0.7) μg/l) than in those with no deficiency (2.8 (SD 0.5) μg/l, p = 0.05), or with functional iron deficiency (2.6 (SD 0.6) μg/l, p<0.001). Children with deficiency of iron stores had a higher mean concentration of sTfR (21.7 (SD 12.5) μg/l) than those with normal iron stores (12.5 (SD 16.2) μg/l, p<0.001), or functional iron deficiency (11.4 (SD 6.0) μg/l, p<0.001). Children with functional iron deficiency had increased mean levels of CRP (126.7 (SD 85.6) mg/l) compared to those with iron stores deficiency (71.9 (SD 74.7) mg/l, p<0.001), or normal iron status (99.8 (SD 70.1) mg/l, p = 0.01).

Ferritin, sTfR and CRP were determined for normal iron and iron deficiency states classified by Gale’s grading method (table 5). Mean log ferritin was higher in those with normal status cases compared to those who were iron deficient. The converse was true for mean sTfR levels. Mean CRP levels were increased in children with normal iron status compared to those classified as iron deficient (p<0.001).

**Table 5:** Mean levels of Log ferritin, sTfR and CRP for iron status categories using Gale’s and Intensive grading methods

Biochemical tests		Gales’ grading method		Intensive grading method			
		Normal	Iron deficiency	Normal	Functional iron deficiency	Iron stores deficiency	Functional and iron stores deficiency
Log ferritin (μg/l)*	Mean (SD) [n]	2.7 (0.5) [89]	1.8 (0.7) [61]	2.8 (0.5) [34]	2.6 (0.6) [44]	1.9 (0.7) [29]	2.1 (0.8) 9
	95% CI	2.6 to 2.8	1.6 to 2.0	2.6 to 2.96	2.4 to 2.8	1.7 to 1.2	1.6 to 2.7
sTfR (μg/ml)†	Mean (SD) [n]	11.8 (10.7) [156]	22.7 (15.9) [86]	12.5 (16.2) [56]	11.4 (6.0) [76]	21.7 (12.5) [44]	27.0 (28.3) 14
	95% CI	10.1 to 13.5	19.4 to 26.1	8.1 to 17.0	10.0 to 12.7	18.0 to 25.4	12.2 to 41.9
CRP (mg/ml)‡	Mean (SD) [n]	122.0 (6.7) [156]	71.9 (8.5) [86]	99.8 (70.1) [56]	126.7 (85.6) [77]	71.9 (74.7) [45]	68.9 (64.9) 13
	95% CI	108.7 to 135.2	55.2 to 88.7	81.5 to 118.2	107.6 to 145.8	50.0 to 93.7	33.6 to 104.2

*Normal >30 μg/l.

†Normal <8.3 μg/ml.

‡Normal <10 mg/ml.

sTfR, soluble transferrin receptor; CRP, C-reactive protein.

## DISCUSSION

The intensive histological grading method attempts to distinguish four different iron states compared to the two categories using Gale’s method. The ability to distinguish states in which there is decreased cellular iron delivery to erythroblasts in the presence of adequate iron stores (termed *functional iron deficiency*), compared to states with limited availability due to lack of available iron in the reticular endothelial system, is of particular importance in areas of high malaria transmission and infection. Although some of the results of the biochemical tests are lacking and some of the aspirates were too poor to examine for iron, this study managed to assess a substantive number of bone marrow aspirates. There were no identifiable reasons for selection bias for the missing results.

Functional iron deficiency, classified using the intensive histological grading method, was based on marrow findings alone. Although levels of CRP, a marker of an acute phase response,^22^ were mostly increased in all children, the finding of significantly raised levels among children with functional iron deficiency supports the hypothesis that these children have anaemia of inflammation. These children appeared also to have adequate iron stores as they had similar levels of ferritin and sTfR to children with normal iron status.

The use of erythroblast iron to assess iron status has been used in other studies^11^ ^18^ and has certain limitations. Marrow smears were counter-stained with Safranin, giving a uniform pink background colour, which makes visualisation of cell types difficult, and hence may affect erythroblast iron assessment. The use of haematoxylin, or May–Grünwald–Giemsa, for counter-staining smears has been recommended as this provides improved cellular detail.^18^ Tham and Macon^23^ demonstrated that use of a silver stain to visualise erythroblast iron was more sensitive than Perls’ stain. However, the precise chemical basis for the silver staining is still unclear.

Some researchers have described erythroblast iron assessment in comparison to simply counting erythroblasts with iron. Baumgartner-Staubli and Beck^11^ developed a “sideroblast score” which assigned an arbitrary value, ranging from 1 to 4 depending on the amount and morphology of iron granules, to each erythroblast with iron. This gave a poor correlation between the sideroblast score and the marrow iron stores assessed using Gale’s grading method.

The Malawi Ministry of Health does not have guidelines on whether to give iron in the management of children with severe anaemia. In practice, most children are prescribed iron, however it is often unavailable and the compliance is poor. This study has shown that approximately 30% of severely anaemic children had deficiency of iron stores (table 3) requiring iron treatment. Methods to identify these children are required in poor-resource settings that are based on simple, less invasive procedures than detailed marrow examination. Additionally this study observes a lower prevalence of deficiency of iron stores among severely anaemic children than controls. This phenomenon is not fully understood, but may support the hypothesis that iron deficiency is associated with decreased risk of infection.^24^

This study of a large sample of bone marrow aspirates demonstrates that using an enhanced bone marrow slide assessment provides more detailed information which allows a more precise iron status classification.

**Take-home messages**

- Differentiation between functional iron deficiency and quantitative deficiency of iron stores is difficult, especially in areas of high infection pressure.
- A new method of grading of iron content of fragments, macrophages and erythroblasts in the bone marrow is able to distinguish between functional and quantitative iron deficiency in anaemic children.
- Severely anaemic Malawian children have less quantitative iron deficiency than controls without severe anaemia.
